# Metabolic engineering of *Escherichia coli* for the production of hydroxy fatty acids from glucose

**DOI:** 10.1186/s12896-016-0257-x

**Published:** 2016-03-08

**Authors:** Yujin Cao, Tao Cheng, Guang Zhao, Wei Niu, Jiantao Guo, Mo Xian, Huizhou Liu

**Affiliations:** CAS Key Laboratory of Biobased Materials, Qingdao Institute of Bioenergy and Bioprocess Technology, Chinese Academy of Sciences, Qingdao, 266101 China; Department of Chemistry, University of Nebraska-Lincoln, Lincoln, NE 68588 USA

**Keywords:** Hydroxy fatty acid, *Escherichia coli*, Fatty acid hydroxylase, Acetyl-CoA carboxylase, Acyl-CoA thioesterase, Acyl-CoA synthetase

## Abstract

**Background:**

Hydroxy fatty acids (HFAs) are valuable chemicals for a broad variety of applications. However, commercial production of HFAs has not been established so far due to the lack of low cost routes for their synthesis. Although the microbial transformation pathway of HFAs was extensively studied decades ago, these attempts mainly focused on converting fatty acids or vegetable oils to their hydroxyl counterparts. The use of a wider range of feedstocks to produce HFAs would reduce the dependence on oil crops and be expected to cut down the manufacturing cost.

**Results:**

In this study, the industrially important microorganism *Escherichia coli* was engineered to produce HFAs directly from glucose. Through the coexpression of the acetyl-CoA carboxylase (ACCase) and the leadless acyl-CoA thioesterase (‘TesA), and knockout of the endogenous acyl-CoA synthetase (FadD), an engineered *E. coli* strain was constructed to efficiently synthesize free fatty acids (FFAs). Under shake-flask conditions, 244.8 mg/L of FFAs were obtained by a 12 h induced culture. Then the fatty acid hydroxylase (CYP102A1) from *Bacillus megaterium* was introduced into this strain and high-level production of HFAs was achieved. The finally engineered strain BL21ΔfadD/pE-A1’tesA&pA-acc accumulated up to 58.7 mg/L of HFAs in the culture broth. About 24 % of the FFAs generated by the thioesterase were converted to HFAs. Fatty acid composition analysis showed that the HFAs mainly consisted of 9-hydroxydecanoic acid (9-OH-C10), 11-hydroxydodecanoic acid (11-OH-C12), 10-hydroxyhexadecanoic acid (10-OH-C16) and 12-hydroxyoctadecanoic acid (12-OH-C18). Fed-batch fermentation of this strain further increased the final titer of HFAs to 548 mg/L.

**Conclusions:**

A robust HFA-producing strain was successfully constructed using glucose as the feedstock, which demonstrated a novel strategy for bioproduction of HFAs. The results of this work suggest that metabolically engineered *E. coli* has the potential to be a microbial cell factory for large-scale production of HFAs.

**Electronic supplementary material:**

The online version of this article (doi:10.1186/s12896-016-0257-x) contains supplementary material, which is available to authorized users.

## Background

The depletion of the earth’s fossil energy resources and global climate change have stimulated us to develop environmentally friendly processes to produce fuels and chemicals. Hydroxy fatty acids (HFAs) are important fine chemicals which have a hydroxyl group in the carbon chain of fatty acids. Due to their unique attributes, HFAs have wide applications in different fields such as surfactants, lubricants, cosmetics or antimicrobials [1, 2]. They are also used as the intermediates for the production of a variety of value-added products [3]. More importantly, HFAs could serve as the precursors for the preparation of the next generation plastics, polyhydroxyalkanoates (PHAs) [4]. PHAs are completely biodegradable and possess good thermoplastic or elastomeric properties. Therefore, PHA bioplastics offer an alternative to conventional petrochemical-derived plastics [5].

Now, HFAs are commercially unavailable due to the lack of low cost routes for their synthesis. Chemical catalysts for specific hydroxylation reactions on the selective carbon atom of the fatty acyl chain are limited [6]. On the other hand, HFAs make up an interesting group of natural compounds among plants, bacteria, yeasts and fungi. A number of microorganisms capable of producing HFAs from fatty acids or vegetable oils have been isolated. For example, *Bacillus pumilus* could hydroxylate oleic acid on the 1, 2, and 3 carbon atoms to produce hydroxy oleic acids [7]. *Candida tropicalis* also excretes HFAs as by-products when cultured on n-alkanes or fatty acids as the carbon source [8]. Enzymes catalyzing the bioconversion of fatty acids to HFAs have been identified as the cytochrome P450 monooxygenases (CYPs). CYPs responsible for the hydroxylation of fatty acids have been cloned from several *Bacillus* species including *B. megaterium* [9], *B. subtilis* [10], *B. anthracis* [11] and *B. cereus* [12]. The CYP102A1 from *B. megaterium* is the most thoroughly studied member of these enzymes. Heterologous expression of this enzyme in *E. coli* indicated that the whole-cell biocatalyst showed the maximum activity to pentadecanoic acid and the resulting products were only 1, 2 and 3 HFAs [13]. This bioconversion has been demonstrated at the 2 L scale fermentor level under oxygen limitation, showing that 12-, 13-, and 14-hydroxypentadecanoic acids can be produced in the g/L range [14]. Recombinant *E. coli* cells harboring another fatty acid hydroxylase P450foxy from the fungus *Fusarium oxysporum* [15] could also convert saturated fatty acids with a chain length of 7–16 carbon atoms to their 1, 2 and 3 hydroxyl derivatives [16].

**Fig. 1:**
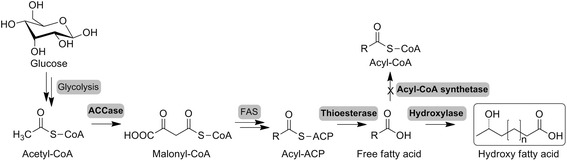
Metabolic pathway from glucose to HFAs in engineered *E. coli*. Glucose is degraded into acetyl-CoA through glycolysis. Acetyl-CoA carboxylase (ACCase) catalyzes the irreversible carboxylation of acetyl-CoA to produce malonyl-CoA. The discrete, monofunctional type II fatty acid synthases (FAS) act on malonyl-CoA to synthesize fatty acyl-ACPs. Then thioesterase hydrolyzes the acyl-ACPs bond to form FFAs. At last, fatty acid hydroxylase transforms FFAs to HFAs. Acyl-CoA synthetase responsible for fatty acid degradation is knocked out to block fatty acids and HFAs degradation

The above studies used fatty acids or their derivatives as the feedstocks for production of HFAs. Compared with the plant oil resources, renewable sugars from biomass are more easily available. In our previous study, we constructed an engineered *E. coli* strain for the direct production of HFAs from glucose through producing free fatty acids (FFAs) by a thioesterase and further converting FFAs to HFAs using a fatty acid hydroxylase [17]. However, production of HFAs of this strain was still too low. Here, the *E. coli* strain was further improved to enhance production of HFAs. The native *E. coli* acetyl-CoA carboxylase (ACCase) and a leadless thioesterase ‘TesA were overexpressed to boost the host cell to produce FFAs. The fatty acid degradation pathway was blocked by disrupting the endogenous acyl-CoA synthetase (FadD). And the FFAs were then converted to HFAs by the fatty acid hydroxylase CYP102A1 (Fig. 1). The finally engineered strain was evaluated under fed-batch conditions and showed a promising perspective for large-scale production of HFAs.

## Results and discussion

### Expression of the recombinant enzymes in E. coli

**Fig. 2:**
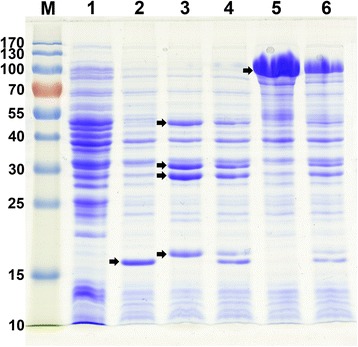
Expression of the recombinant enzymes in engineered *E. coli*. Lane M, prestained protein ladder; lane 1, strain BL21 star(DE3) harboring pET28a; lane 2, strain BL21 star(DE3) harboring pE-‘tesA; lane 3, strain BL21 star(DE3) harboring pA-acc; lane 4, strain BL21 star(DE3) harboring both pE-‘tesA and pA-acc; lane 5, strain BL21 star(DE3) harboring pE-A1; lane 6, strain BL21 star(DE3) harboring both pE-A1’tesA and pA-acc. The positions corresponding to the overexpressed proteins are indicated by an arrow

With the aim to express the ACCase, ‘TesA and CYP102A1 enzymes, we cloned the coding regions of the corresponding genes into plasmids pACYCDuet-1 pET28a, or pET30a under the control of T7 promoter. To verify the expression levels of the recombinant proteins, *E. coli* BL21(DE3) was transformed by the expression vectors pE-‘tesA, pA-acc, pE-A1, pE-A1’tesA or a combination of these vectors. The resulting recombinant strains were grown in liquid LB medium followed by IPTG induction. The bacterial cells were collected and subjected to ultrasonication, and the lysates were then analyzed by SDS-PAGE. Figure 2 showed the gel electrophoresis patterns of samples from different recombinant strains. We noted distinct bands of the expected sizes from protein extracts of the recombinant strains compared with the control strain harboring pET28a. SDS-PAGE analysis of the recombinant strain carrying pE-‘tesA revealed the band of the molecular weight 20.5 kDa (lane 2), which corresponded to the size of the leadless ‘TesA [18]. Strain BL21/pA-acc gave all the bands of the four subunits of ACCase (lane 3). The recombinant strain expressing both ‘tesA and ACCase displayed the protein bands for the two genes (lane 4). Strain BL21/pE-A1 showed a band corresponding to the molecular weight of CYP102A1 (119 kDa, lane 5) [19]. Unlike the membrane-bound CYPs in eukaryotic systems, the bacterial CYPs usually exist in a soluble form [20]. Therefore, the CYP102A1 enzyme could function properly in the cytoplasm of the recombinant cells. The finally engineered strain BL21/pE-A1’tesA&pA-acc gave all the bands of the recombinant proteins (lane 6).

### Production of FFAs by engineered E. coli

CYP102A1 catalyzes the hydroxylation of FFAs to form HFAs. Therefore, the first step to produce HFAs from glucose is to create an intracellular FFAs pool. Many studies have been performed to synthesize FFAs using *E. coli* [21]. In this research, we constructed a recombinant *E. coli* strain efficiently producing FFAs mainly from three aspects. To increase the cellular FFAs level of *E. coli*, the leadless native thioesterase ‘TesA was overexpressed in strain BL21 star(DE3). About 108.5 mg/L of FFAs were produced after being induced for 12 h, which is similar to previous studies [22]. To enhance the precursor supply for fatty acids biosynthesis, the ACCase, which catalyzes the irreversible carboxylation of acetyl-CoA [23], was further coexpressed with ‘TesA. Shake-flask fermentation of strain BL21/pE-‘tesA&pA-acc accumulated up to 188.6 mg/L of FFAs in the culture. To eliminate fatty acid degradation, the acyl-CoA synthetase (FadD) participating in the β-oxidation pathway [24] was knocked out, resulting in strain BL21ΔfadD. Then the recombinant plasmids pE-‘tesA and pA-acc were co-transformed into this strain. The engineered strain BL21ΔfadD/pE-‘tesA&pA-acc was evaluated for production of FFAs and 244.8 mg/L of FFAs were synthesized, about 2.3-fold increase when compared with strain BL21/pE-‘tesA. Gas chromatography - mass spectrometry (GC-MS) analysis (Additional file 1) showed that FFAs in these strains mainly consisted of C8:0, C10:0, C12:1, C12:0, C14:1, C14:0, C16:1, C16:0, and C18:1 fatty acids, with C14, C16 and C18 fatty acids as the dominant constitutes (Table 1).

**Table 1:** FFAs composition produced by different engineered strains

Strains	C8:0	C10:0	C12:0	C12:1	C14:0	C14:1	C16:0	C16:1	C18:1	Total
BL21/pE-‘tesA	1.36 (1.3 %)	1.65 (1.5 %)	8.44 (7.8 %)	5.47 (5.0 %)	10.64 (9.8 %)	5.88 (5.4 %)	42.0 (38.7 %)	12.4 (11.4 %)	20.7 (19.1 %)	108.5
BL21/pE-‘tesA&pA-acc	2.15 (1.2 %)	2.86 (1.5 %)	15.1 (8.1 %)	9.6 (5.1 %)	18.9 (10.1 %)	10.2 (5.5 %)	71.3 (38.2 %)	21.3 (11.4 %)	35.2 (18.9 %)	186.6
BL21ΔfadD/pE-‘tesA&pA-acc	3.64 (1.5 %)	3.89 (1.6 %)	20.4 (8.3 %)	13.1 (5.3 %)	25.2 (10.3 %)	13.2 (5.4 %)	90.3 (36.9 %)	28.3 (11.6 %)	46.8 (19.1 %)	244.8

The unit value for the fatty acids was mg/L

### Identification of HFAs from the CYP102A1 expressing strain

As shown above, FFAs of different chain length and saturation were efficiently produced by the recombinant strains. In order to convert these FFAs to their hydroxyl counterparts, the fatty acid hydroxylase CYP102A1 was further coexpressed in these FFA overproducing strains. To identify the HFAs produced by CYP102A1, the extracts from the culture broth of strain BL21/pE-A1’tesA coexpressing ‘tesA and CYP102A1 were derivatized to their methyl esters and then analyzed by GC-MS. The mass spectrums of the hydroxy fatty acid methyl esters (HFAMEs) prepared from a 12 h - induced culture were shown in Additional file 1. Qualitative analysis was performed using a National Institute of Standards and Technology (NIST) - library search program. Four types of HFAs, 9-hydroxydecanoic acid methyl ester (9-OH-C10), 11-hydroxydodecanoic acid (11-OH-C12), 10-hydroxyhexadecanoic acid (10-OH-C16) and 12-hydroxyoctadecanoic acid (12-OH-C18), were detected in this strain. It has been reported that the fatty acid hydroxylase CYP102A1 has a broad substrate specificity [20]. This enzyme could catalyze the hydroxylation of saturated or unsaturated fatty acids with a chain length of 12–22 carbons [25]. The hydroxylation always occurred in the subterminal position while the terminal methyl group of these substrates was never hydroxylated. The hydroxyl position could also be altered by rational mutagenesis of specific amino acid sites [26]. Here we further identify 10-OH-C16 and 12-OH-C18 from the mixture of hydroxylated products in addition to the subterminal ω-HFAs. It seems that CYP102A1 could oxidate the double bonds of the two kinds of unsaturated fatty acid, palmitoleic acid (C16:1Δ9) and cis-vaccenic acid (C18:1Δ11), and generate 10-OH-C16 and 12-OH-C18 HFAs. C16 or C18 HFAs at the subterminal positions were not identified in our engineered strain. Although these FFAs made up the major portion in the total fatty acid profiles, the catalytic activity of CYP102A1 towards them was much lower. This result was in accordance with many previous studies. CYP102A1 was more efficient toward medium-chain fatty acids and the catalytic activity of this enzyme decreased when the fatty acid chain length was greater than 15 [13, 17]. Therefore, we cannot detect any C16 or C18 HFAs at the subterminal positions from the CYP102A1 overexpressing strain.

Unlike the fungal fatty acid hydroxylases [27], the bacterial CYP102A1 does not act on the terminal position of the fatty acid chain. Therefore, the hydroxyl products of this enzyme could not be degraded by the host’s endogenous enzymes, such as the fatty alcohol oxidase [28]. These HFAs were stable in the fermentation broth and could accumulate without deleting the ω-HFAs degradation enzymes. In addition, CYP102A1 is a self-sufficient fatty acid hydroxylase [29]. It consists of a heme-binding domain and a FMN/FAD-containing domain, and catalyzes the electron transfer from NADPH, via FAD, FMN, and heme, to O_2_, resulting in the formation of a hydroxyl group on carbon atoms without the help of other enzymes [30]. This is different from many fatty acid hydroxylases which require the ferredoxin (Fdx) reductase domain to obtain reducing equivalents from NADPH [31]. Thus, the CYP102A1-based HFA-producing system would be much easier to be operated.

### Evaluation of HFAs producing ability of different strains under shake-flasks conditions

**Fig. 3:**
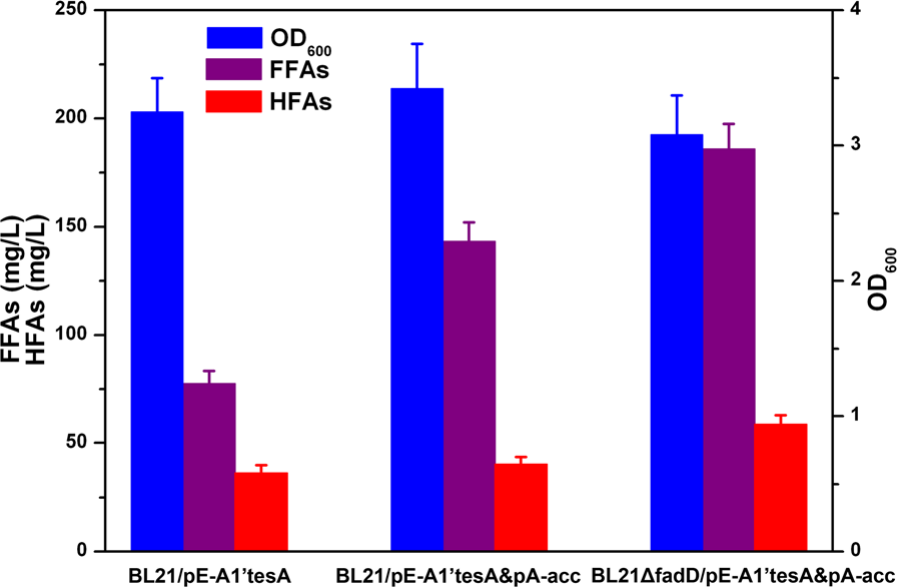
Comparison of HFAs production of several different strains under shake-flask conditions. Data were obtained after each strain was induced for 12 h in liquid LB medium supplemented with 20 g/L glucose. BL21/pE-A1’tesA, strain BL21 star(DE3) expressing *B. megaterium* CYP102A1 and native *E. coli* ‘tesA; BL21/pE-A1’tesA&pA-acc, further overexpressing native *E. coli* ACCase; BL21ΔfadD/pE-A1’tesA&pA-acc, knockout of native *fadD* gene while coexpressing the three enzymes

To investigate the supply of FFAs on HFAs production, different *E. coli* strains including BL21/pE-A1’tesA, BL21/pE-A1’tesA&pA-acc and BL21ΔfadD/pE-A1’tesA&pA-acc were cultivated under shake-flask conditions. Fatty acids produced by these strains were extracted, derivatized and analyzed by GC-MS. The quantities of FFAs and HFAs were determined by comparison of the chromatographic peak areas with the internal standard (C20 or 12-OH-C12). Cell density, FFAs and HFAs accumulated in the fermentation broth of different recombinant strains were calculated and shown in Fig. 3. It could be seen that all the three recombinant strains grew to a similar OD_600_ after 12 h induction (about 3.0-3.5). Strain BL21/pE-A1’tesA produced 77.5 mg/L of FFAs and 36.5 mg/L of HFAs. When ACCase was overexpressed, the final titer of FFAs and HFAs reached 143.4 mg/L and 40.3 mg/L, respectively. Production of FFAs was greatly improved in this strain, but there was only a slight increase in production of HFAs. The FFAs accumulated seemed not to be efficiently converted to HFAs by the fatty acid hydroxylase. When *E. coli* native *fadD* gene was knocked out, the finally engineered strain BL21ΔfadD/pE-A1’tesA&pA-acc showed an enhanced ability to produce HFAs. The titer of HFAs reached 58.7 mg/L, which is 1.6-fold to the original strain. Compositions of HFAs in these strains were shown in Table 2. 11-Hydroxydodecanoic acid and 12-hydroxyoctadecanoic acid made up the major HFAs constituents. The deletion of *fadD* could block both fatty acids and degradation of HFAs [13]. Thus, production of FFAs and HFAs was increased in this strain. The productivity of HFAs per cell dry weight (CDW) of strain BL21ΔfadD/pE-A1’tesA&pA-acc reached 44.3 mg/gCDW (1 OD_600_ = 0.43 gCDW). The enhancement of production of FFAs was much greater than production of HFAs along with the introduction of ACCase and knockout of *fadD*. Only 24 % of the FFAs were converted to HFAs in this finally engineered strain. These results indicated that the rate-limiting step for HFAs production was the fatty acid hydroxylase CYP102A1 [32]. We can expect to achieve a higher titer of HFAs by improving the efficiency of this enzyme.

**Table 2:** HFAs composition produced by different engineered strains

Strains	9-OH-C10	10-OH-C16	11-OH-C12	12-OH-C18	Total
BL21/pE-A1’tesA	4.17 (11.4 %)	3.31 (9.1 %)	18.7 (51.1 %)	10.4 (28.4 %)	36.5
BL21/pE-A1’tesA&pA-acc	4.80 (11.9 %)	3.91 (9.7 %)	20.2 (50.1 %)	11.4 (28.3 %)	40.3
BL21ΔfadD/pE-A1’tesA&pA-acc	7.16 (12.2 %)	5.87 (10.0 %)	29.3 (49.8 %)	16.4 (28.0 %)	58.7

The unit value for the HFAs was mg/L

### HFAs production at the fermentor scale

**Fig. 4:**
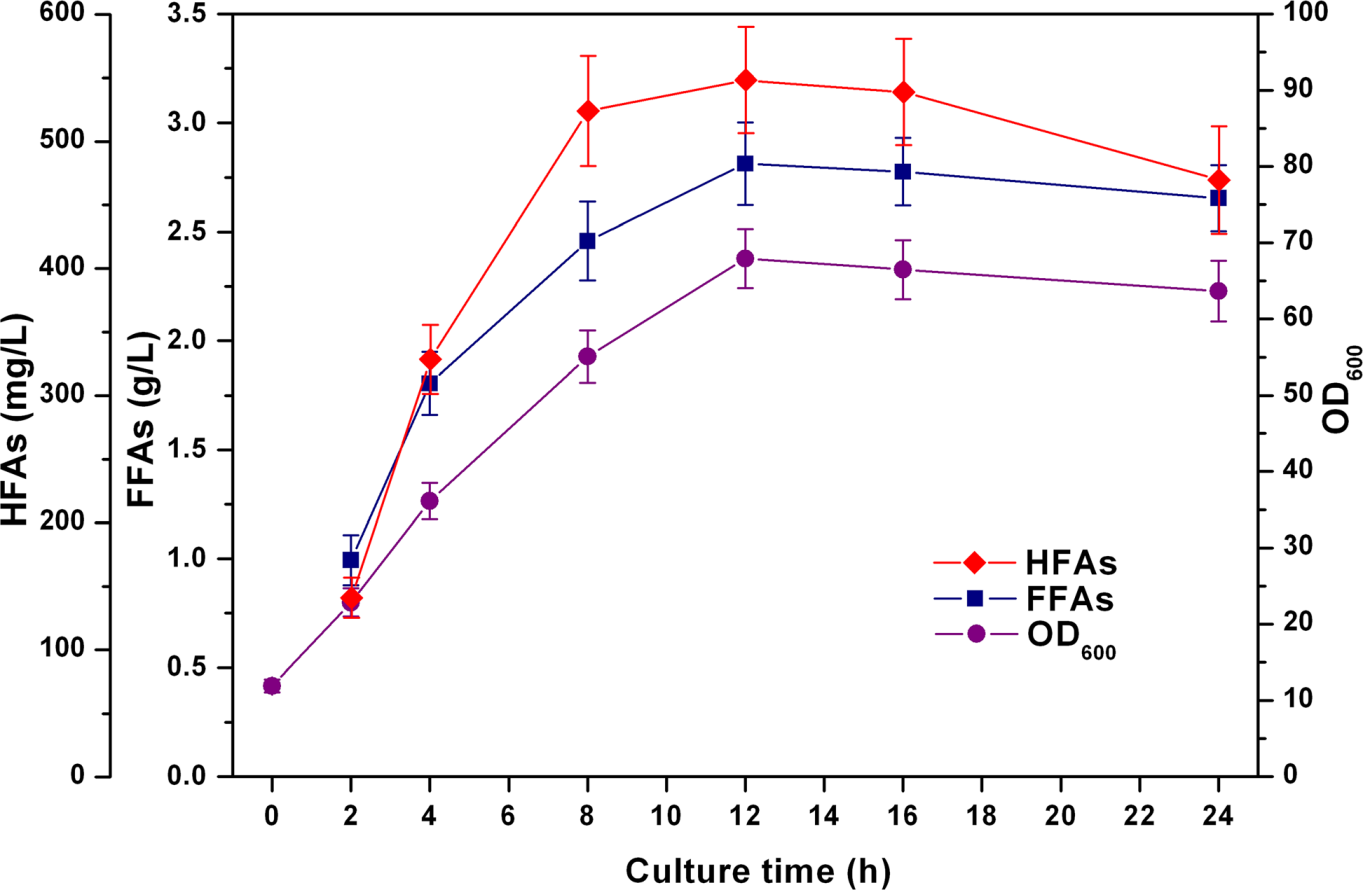
Time courses of cell density (OD_600_), FFAs and HFAs production during fed-batch culture of the finally engineered strain BL21ΔfadD/pE-A1’tesA&pA-acc. Cultivation was conducted in a 5 L fermentor with an initial volume of 2 L of rich growth medium

Microbial production of HFAs is currently achieved using the bacteria *Pseudomonas* sp. [33] or nonconventional yeasts *Candida* sp. [34] that produce selective CYPs as the hosts. Compared with these strains, *E. coli* has many advantages such as a clear genetic background, high convenience to be genetically modified, and good growth properties with low nutrient requirements [35]. Here, we tested our recombinant *E. coli* strain using high-density fermentation strategy. Based on the results obtained by the shake-flask cultivations, the finally engineered *E. coli* strain BL21ΔfadD/pE-A1’tesA&pA-acc was cultured in a 5 L-scale laboratory fermentor. Cell density, residual glucose concentration and products accumulation were monitored over the course of fed-batch fermentation. Figure 4 shows the time profiles of cell density and production of HFAs during 24 h fed-batch fermentation. The bacteria grew very fast at the first 12 h post-induction to an OD_600_ of approximate 70. FFAs and HFAs also accumulated rapidly in the culture broth. The highest production of FFAs and HFAs were obtained after 12 h induction, that is, 2.81 g/L and 548 mg/L. The volumetric productivities of FFAs and HFAs were 234 mg/(L · h) and 45.7 mg/(L · h), respectively. Both of the titers of FFAs and HFAs decreased to some extent in the following fermentation processes.

Compared with the HFA-producing process using fatty acids as the feedstock [1, 36–38], the current production and yield obtained by this engineered *E. coli* strain is still too low. This might be due to that these processes used quite different mechanisms to synthesize HFAs. The double bond hydratases were employed in previous work directly acting on unsaturated fatty acids to generate HFAs. The catalytic activity of the hydratases was more efficient than the P450 monooxygenase used in this study, leading to much higher productivity and yield. However, the use of fatty acids or plant oils increases the raw material cost since they are more expensive than glucose and other sugars. These carbohydrates have the potential to be manufactured from the easily available lignocellulosic biomass resources. The biotransformation of fatty acids also needed to first grow the cells with glucose or other carbon sources. The yield was overestimated for neglecting the consumption of the carbon sources. In addition, the yeast-based HFAs-producing strains always take several days to reach the maximum titer, while the whole fermentation process only requires less than 24 h for this engineered strain.

The production and yield of HFAs in the present study could be enhanced from several aspects. Biosynthesis of FFAs is the first rate-limiting step in our HFA-producing system. Fatty acid biosynthesis from glucose requires carbon fluxes through glycolysis to generate pyruvate which is further dehydrogenated to acetyl-CoA. Acetyl-CoA is then carboxylated to form malonyl-CoA, the precursor of the bacterial type II fatty acid synthases [39]. Fatty acyl-ACPs of different chain-length were finally cleaved by thioesterases into FFAs. Numerous effects have been conducted to improve the ability of *E. coli* to synthesize FFAs, but the highest titer of FFAs achieved up to now was roughly 9 g/L [40]. To obtain an even higher production of HFAs, the FFAs pool must be further increased. Fatty acid hydroxylase is another key enzyme for production of HFAs. Although the CYP102A1 enzyme has many excellent attributes, its catalytic activity is much lower than many other hydroxylases, e.g., lipoxygenase, hydratase and diol synthase [41]. Therefore, the use of more efficient fatty acid hydroxylases in our producing system would be helpful to improve production of HFAs.

## Conclusions

In this study, a robust HFA-producing *E. coli* strain was successfully constructed. Four distinct genetic alterations targeted at the HFA metabolic pathways were introduced into the host strain BL21 star(DE3), including knockout of the endogenous *fadD* gene, which encodes the acyl-CoA synthetase, to block fatty acid β-oxidation; overexpression of native *E. coli* ACCase to enhance the supply of malonyl-CoA, the precursor for fatty acid biosynthesis; overexpression of a leadless thioesterase ‘TesA to render the host releasing FFAs; and further introducing a hydroxylase CYP102A1 to hydroxylate the FFAs into HFAs. Under fed-batch conditions, up to 548 mg/L of HFAs were produced by the finally engineered strain BL21ΔfadD/pE-A1’tesA&pA-acc. The volumetric productivity of HFAs reached 45.7 mg/(L · h). Although the current production of this work is far from industrial application, it opens the door to employing the enormous power of metabolic engineering in this experimentally friendly organism for HFAs biosynthesis. This engineered *E. coli* would give some implication to industrial-scale production of HFAs in the future.

## Methods

### Bacterial strains and plasmids construction

A list of bacterial strains and recombinant plasmids was presented in Table 3. *E. coli* DH5α was used for gene cloning and *E. coli* BL21 star(DE3) was used as the host for the expression of the recombinant proteins. The chromosomal *fadD* gene of strain BL21 star(DE3) responsible for fatty acid degradation was knocked out using the Red recombination strategy in a previous study, resulting strain BL21ΔfadD [42].

**Table 3:** Strains and plasmids used in this study

Strains or plasmids	Genotype/Description	Sources
Strains		
*E. coli* BL21 star(DE3)	*F* ^*−*^ *ompT hsdS*_*B*_ (r_B_^−^ m_B_^−^) *gal dcm rne131* (DE3)	Invitrogen
*E. coli* BL21 star(DE3) ΔfadD	Knockout of *fadD* encoding acyl-CoA synthetase	[42]
Plasmids		
pET28a(+)	Kan^r^ *oripBR322 lacI* ^*q*^ *T7p*	Novagen
pET30a(+)	Kan^r^ *oripBR322 lacI* ^*q*^ *T7p*	Novagen
pACYCDuet-1	*Cm* ^*r*^ *oriP15A lacI* ^*q*^ *T7p*	Novagen
pE-‘tesA	pET30a(+) harboring *E. coli ‘tesA*	This study
pE-A1	pET28a(+) harboring *B. megaterium CYP102A1*	This study
pE-A1’tesA	pET28a(+) harboring both *E. coli ‘tesA and B. megaterium CYP102A1*	This study
pA-acc	pACYCDuet-1 harboring *E. coli ACCase*	[43]

Primers used for plasmids construction was provided in Table 4. The four subunits of native *E. coli* acetyl-CoA carboxylase were cloned into a single expression vector pACYCDuet-1, resulting pA-acc in another study [43]. The *CYP102A1* gene [GenBank: J04832] was PCR amplified from *B. megaterium* ATCC 14581 genomic DNA and cloned into the restriction sites *Nco*I/*Eco*RI of vector pET28a, resulting pE-A1. The *‘tesA* gene [GenBank: EG11542] (encoding a leadless version of native *E. coli* thioesterase I without the N-terminal 26 amino acids) was amplified from *E. coli* K12 genome and cloned into the restriction sites *Nde*I/*Bgl*II of vector pET30a, resulting pE-‘tesA. Then PCR reaction was performed using pE-‘tesA as the template and a primer pair that allowed the amplification of the T7 promoter sequence along with the *‘tesA* structural gene. The PCR product T7’tesA was then cloned into pE-A1 between *Eoc*RI and *Xho*I sites to create pE-A1‘tesA, which was used for the coexpression of the two genes. Successful gene cloning was verified by colony PCR, restriction mapping and direct nucleotide sequencing.

**Table 4:** PCR primers designed for plasmids construction

Oligonucleotide primers	Sequences
‘tesA_ F_NdeI	GGAATTC**CATATG**GCGGACACGTTATTGATTCTGGG
‘tesA_R_BglII	GA**AGATCT**TATGAGTCATGATTTACTAAAGGC
A1_ F_NcoI	CATG**CCATGG**GCATGACAATTAAAGAAATGCCTCAG
A1_ R_EcoRI	CCG**GAATTC**TTACCCAGCCCACACGTCTTTTG
T7'tesA_F_EcoRI	CCG**GAATTC**TAATACGACTCACTATAGGGG
T7′tesA_R_XhoI	CCG**CTCGAG**TTATGAGTCATGATTTACTAAAGGC

### Media and culture conditions

Luria-Bertani (LB) medium (10 g/L tryptone, 5 g/L yeast extract, 10 g/L NaCl) was used for DNA manipulation, protein expression and shake-flasks cultivation. Rich growth medium (20 g/L tryptone, 10 g/L yeast extract, 5 g/L NaCl and 5 g/L K_2_HPO_4_ · 3H_2_O) was used for fermentor-scale cultivation. MgSO_4_ (0.12 g/L) and trace elements (1 ml per liter, 3.7 g/L (NH_4_)_6_Mo_7_O_24_ · 4H_2_O, 2.9 g/L ZnSO_4_ · 7H_2_O, 24.7 g/L H_3_BO_3_, 2.5 g/L CuSO_4_ · 5H_2_O, 15.8 g/L MnCl_2_ · 4H_2_O) were autoclaved or filter-sterilized separately and added prior to initiation of the fermentation. 50 mg/L of kanamycin or 34 mg/L of chloramphenicol were supplemented when necessary. Under shake-flask conditions, the bacterial cultures were first grown at 37 °C and 180 rpm. 0.5 mM of isopropyl-β-D-thiogalactopyranoside (IPTG) was added at an OD_600_ of about 0.6 to induce the expression of recombinant proteins and production of HFAs. Then the culture temperature was shifted to 30 °C after adding the inducer.

### Protein expression and SDS-PAGE analysis

Recombinant *E. coli* strains harboring pE-A1, pE-‘tesA, pE-A1’tesA, pA-acc or the combination of these plasmids were induced for 4 h to express the recombinant proteins. Then cells were collected from 1.5 ml of bacterial cultures by centrifugation and resuspended in 50 mM of Tris–HCl buffer (pH 8.0). Cell pellets were disrupted using a probe-type sonicator (VCX130, Sonics, USA) at 4 °C. The resulting crude extracts were centrifuged and the supernatants with the soluble proteins were recovered, mixed with equal volume of 2× sodium dodecyl sulfate (SDS) sample buffer, heated at 100 °C for 10 min and then analyzed by SDS-polyacrylamide gel electrophoresis (PAGE) according to a standard procedure. Protein bands were visualized with Coomassie Brilliant Blue staining.

### Fed-batch fermentation

For large-scale production of HFAs, fed-batch cultures were carried out in a Biostat B plus MO5L fermentor (Sartorius Stedim Biotech GmbH, Germany) containing 2 L of rich growth medium. 50 ml of inoculum was prepared by incubating the culture in shake flasks at 37 °C overnight. After inoculation, the fermentation was first operated in a batch mode and the control settings were: 37 °C, pH 7.0, airflow at 2 L/min and stirring speed at 400 rpm. The dissolved oxygen (DO) was kept above 20 % by associating with the stirring speed. After the initial glucose was nearly exhausted, fed-batch mode was commenced by feeding a concentrated glucose solution (65 %) at appropriate rates to maintain the residual glucose at a low level. When OD_600_ reached about 12, 0.5 mM of IPTG was used to induce recombinant proteins expression and production of HFAs. Then the culture temperature was switched to 30 °C. Samples of fermentation broth were taken at appropriate intervals to determine cell density, residual glucose, production of FFAs and HFAs.

### Fatty acids and HFAs extraction and the corresponding methyl esters preparation

To extract the FFAs and HFAs from the fermentation broth, the culture broth was acidified with 6 M hydrochloric acid to pH < 2. Eicosanoic acid (C20), 10-hydroxydecanoic acid methyl ester (10-OH-C10) or 12-hydroxydodecanoic acid (12-OH-C12) from a 50 mg/mL stock solution in ethyl acetate were added to the culture broth before extraction to serve as the internal standards. The acidic materials were extracted with equal volume of ethyl acetate. The collected organic layer was evaporated with nitrogen and then the extracts were exposed to sulfuric acid/methanol (1:99, by volume) at 70 °C for 1 h to generate fatty acids or HFAs methyl esters (FAMEs or HFAMEs). The FAMEs and HFAMEs were then extracted with n-hexane.

### Analytical methods

Cell growth of the *E. coli* culture in shake-flasks or fermentors was monitored by determining the optical density at 600 nm (OD_600_) of appropriate dilutions using an UV–vis Spectrophotometer (Cary 50, Varian, USA).

The concentration of residual glucose was quantified by a glucose oxidase-peroxidase assay using an SBA-40D Biological Sensing Analyzer (Biology Institute of Shandong Academy of Sciences, China).

The resulting FAMEs and HFAMEs were analyzed an Agilent Trace GC 7890A system coupled to a quadrupole detector (5975C). The GC was equipped with a 30 m HP-5 ms column (internal diameter 0.25 mm, film thickness 0.25 μm), an ion source temperature of 220 °C and EI ionization at 70 eV. The method used a 10:1 split ratio and nitrogen as carrier gas with a linear velocity of 1 ml/min. The temperature program was an initial hold at 100 °C for 2 min, ramping at 10 °C per min to 200 °C followed by a temperature gradient of 5 °C per min to 280 °C and a final hold at 280 °C for 5 min. Since authentic standards for the HFAMEs were not available, these compounds were identified by searching the NIST Mass Spectral Library [44]. Quantification of FFAs and HFAs were performed by comparison to the internal standard.

## Availability of data and materials

The dataset supporting the conclusions of this article is included within the article (and its additional file).

## Additional file

Additional file 1: Identification of the FFAs and HFAs by GC-MS analysis. The structures of FFAs and HFAs were matched by searching a standard NIST library. (DOC 748 kb)
