# Supplementary material for: Metabolic engineering of Escherichia coli for the production of hydroxy fatty acids from glucose
Source: BMC Biotechnol. 2016 Mar 8;16:26. doi: 10.1186/s12896-016-0257-x (PMC4782510; doi:10.1186/s12896-016-0257-x)
Supplement: Additional file 1: — Identification of the FFAs and HFAs by GC-MS analysis. The structures of FFAs and HFAs were matched by searching a standard NIST library. (DOC 748 kb) [file 12896_2016_257_MOESM1_ESM.doc]

**Additional file 1**

**Identification of the FFAs and HFAs by GC-MS analysis.** The structures of FFAs and HFAs were matched by searching a standard NIST library.


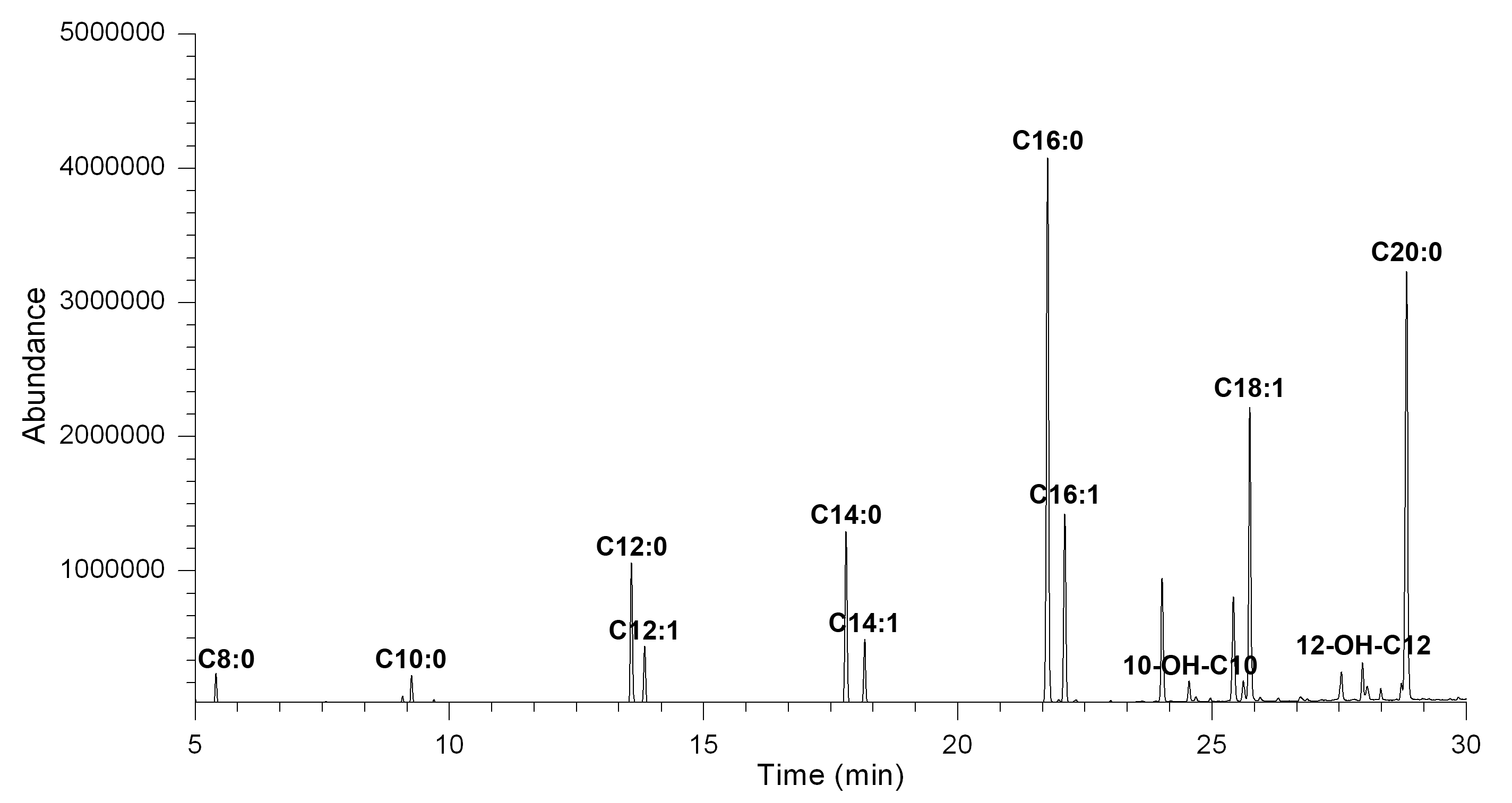


GC chromatogram of FFAs and HFAs


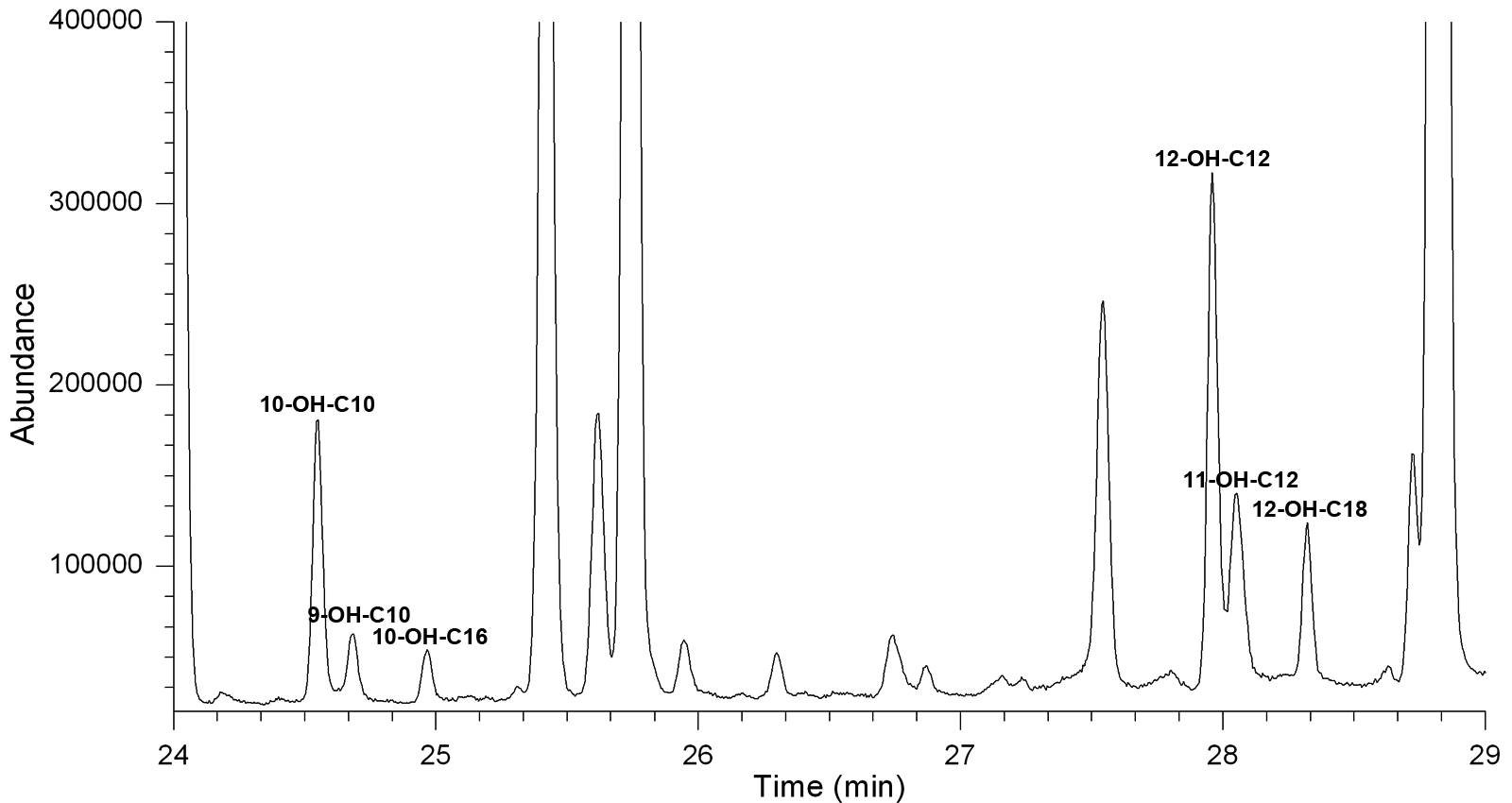


Detailed GC chromatogram of HFAs from 24 min to 29 min


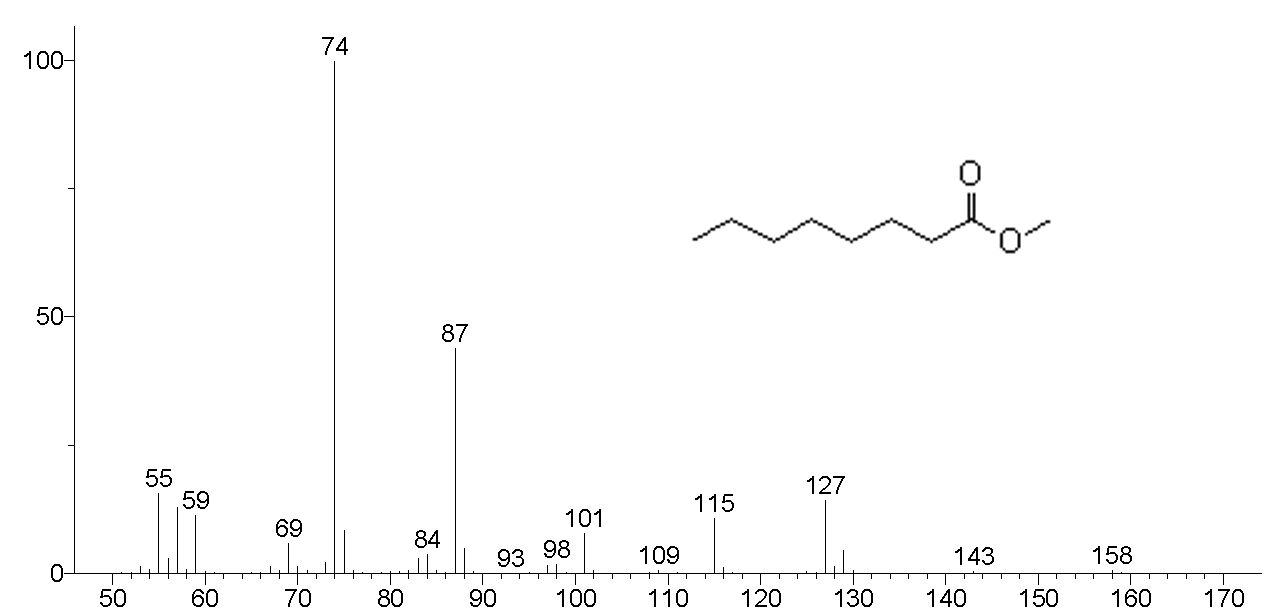


Mass spectrum of octanoic acid methyl ester (C8:0)


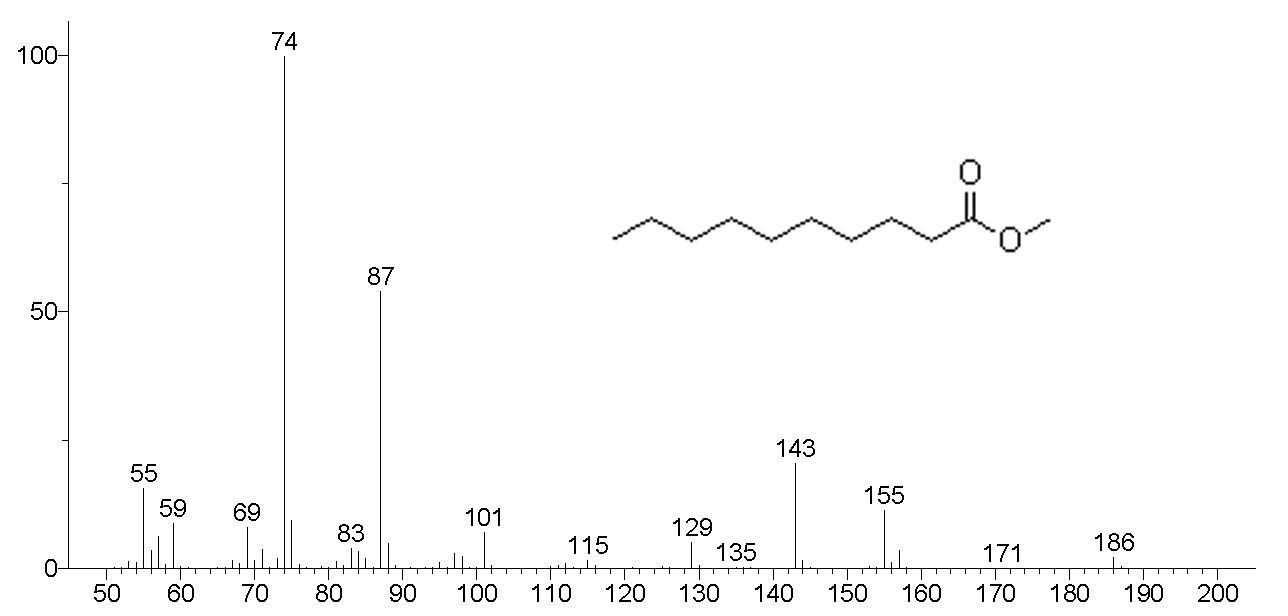


Mass spectrum of capric acid methyl ester (C10:0)


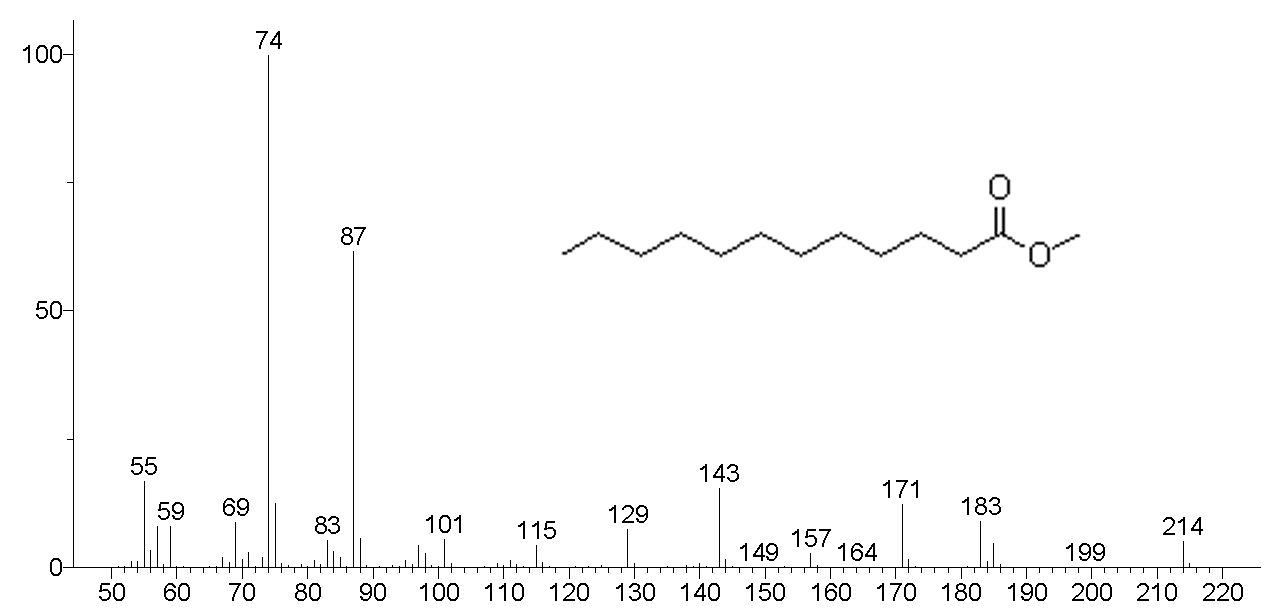


Mass spectrum of lauric acid methyl ester (C12:0)


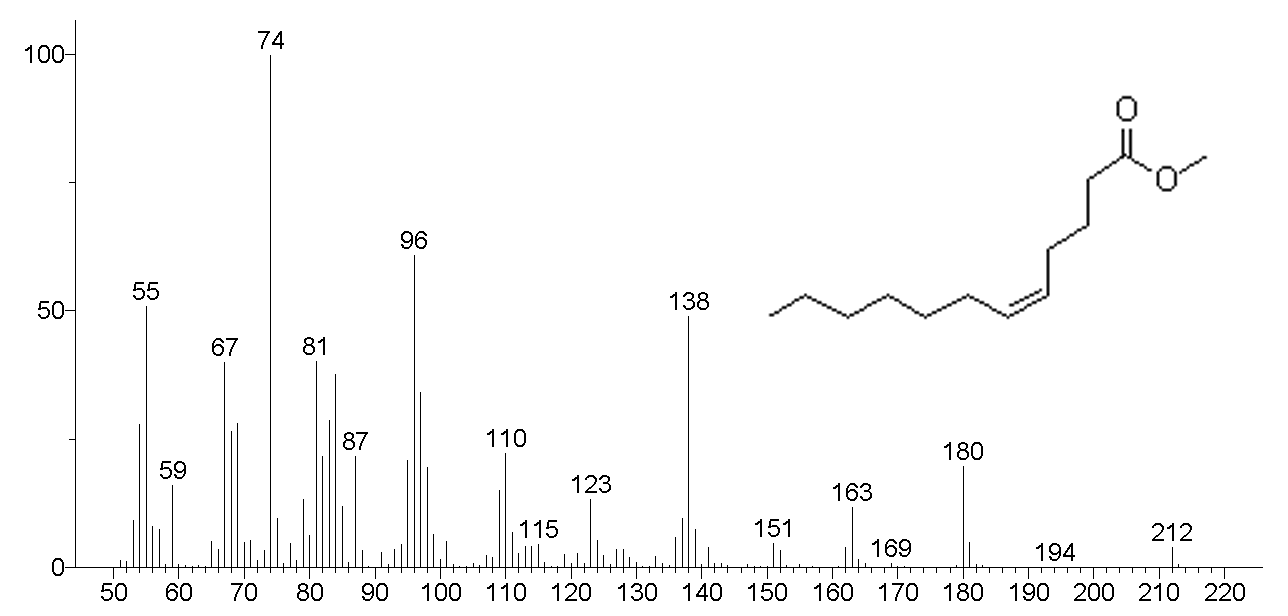


Mass spectrum of *cis*-5-dodecenoic acid methyl ester (C12:1)


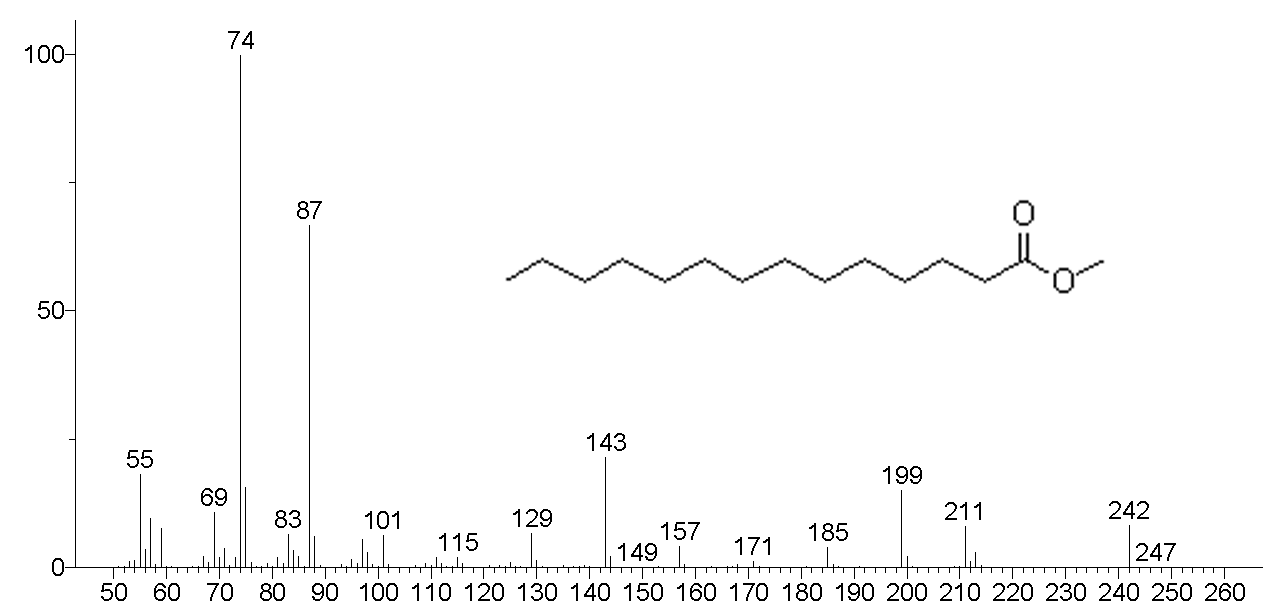


Mass spectrum of myristic acid methyl ester (C14:0)


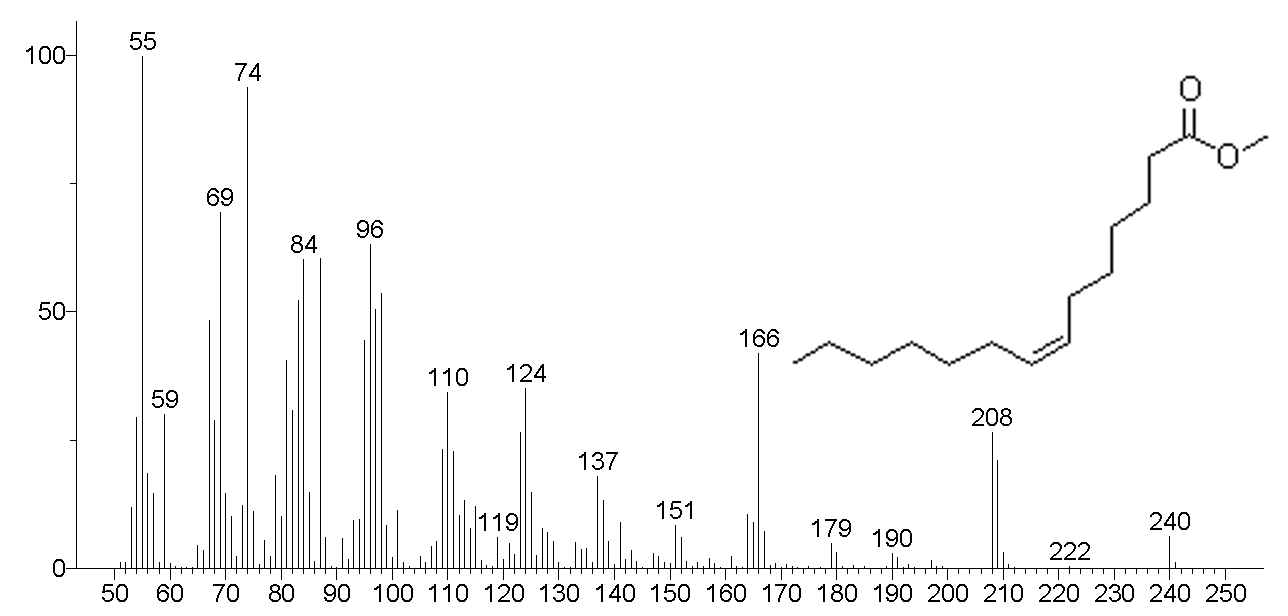


Mass spectrum of *cis*-7-tetradecenoic acid methyl ester (C14:1)


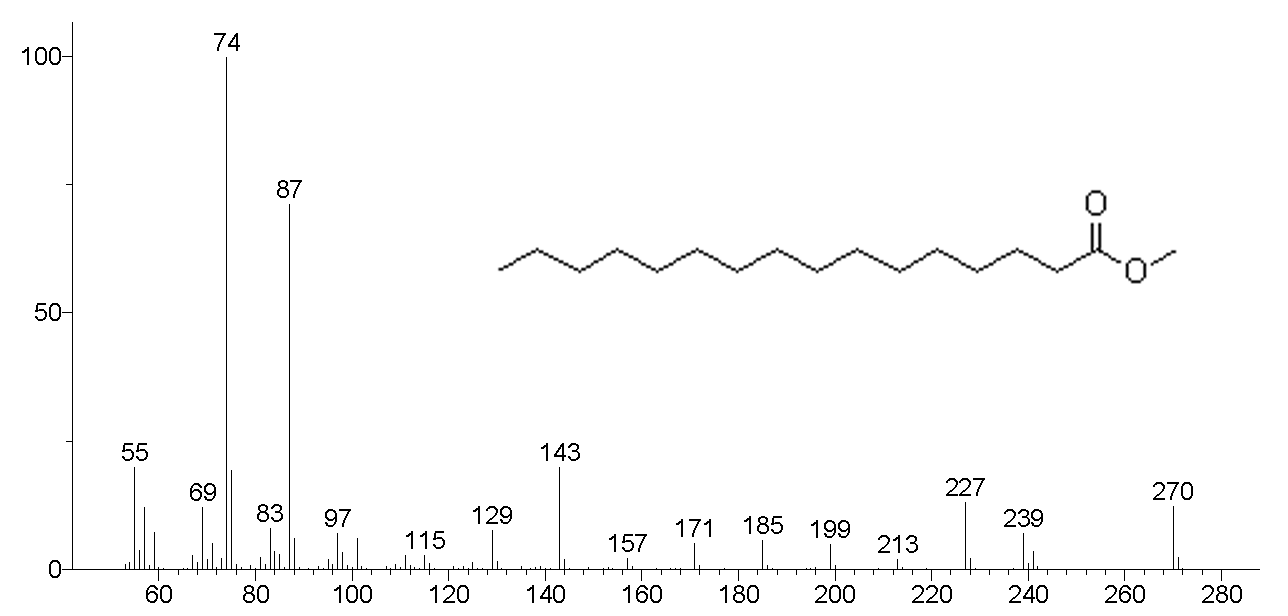


Mass spectrum of palmitic acid methyl ester (C16:0)


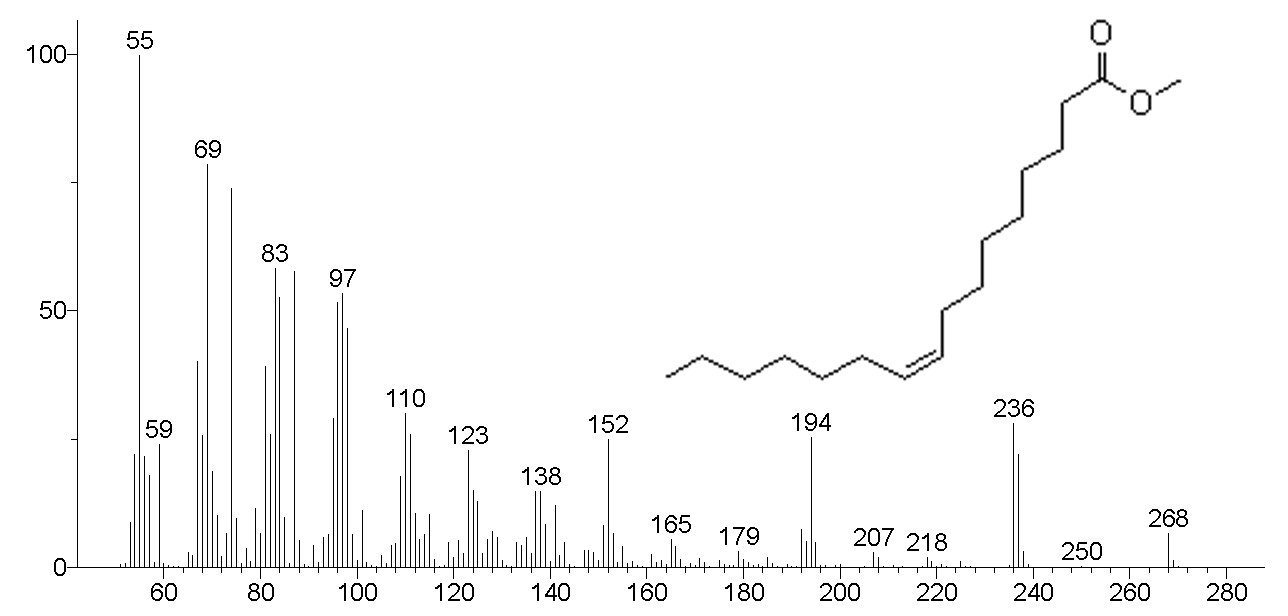


Mass spectrum of palmitoleic acid methyl ester (C16:1)


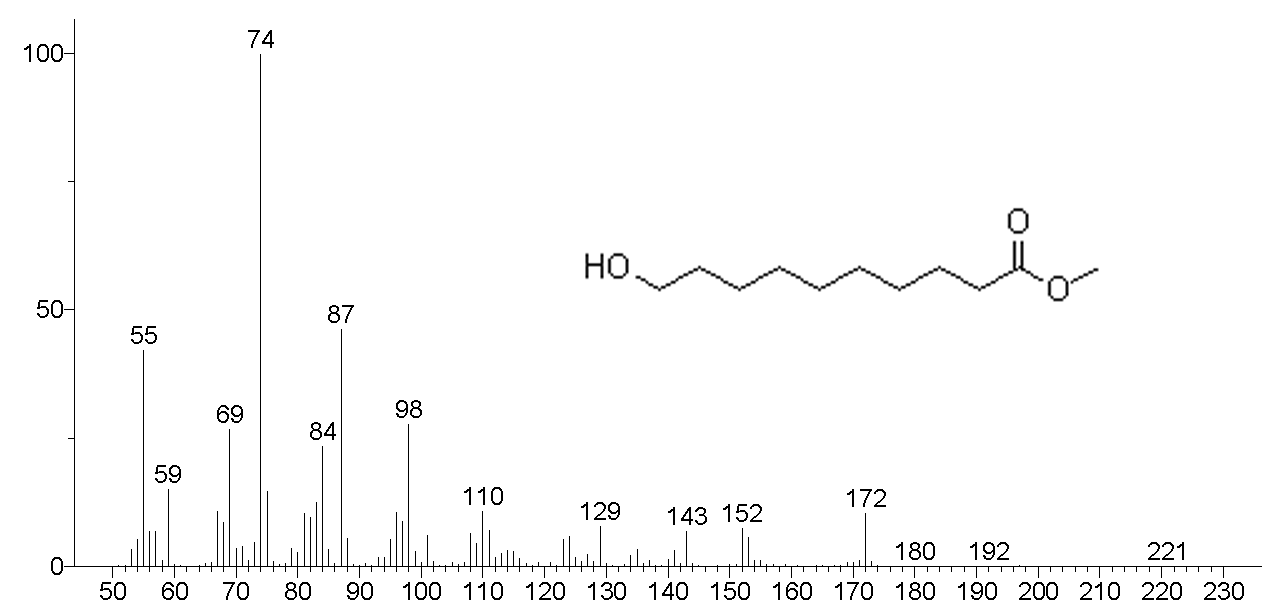


Mass spectrum of 10-hydroxydecanoic acid methyl ester (10-OH-C10)


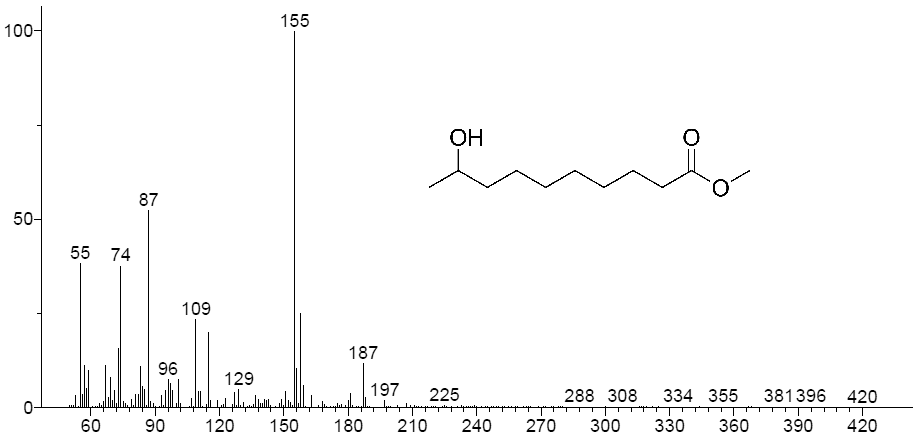


Mass spectrum of 9-hydroxydecanoic acid methyl ester (9-OH-C10)


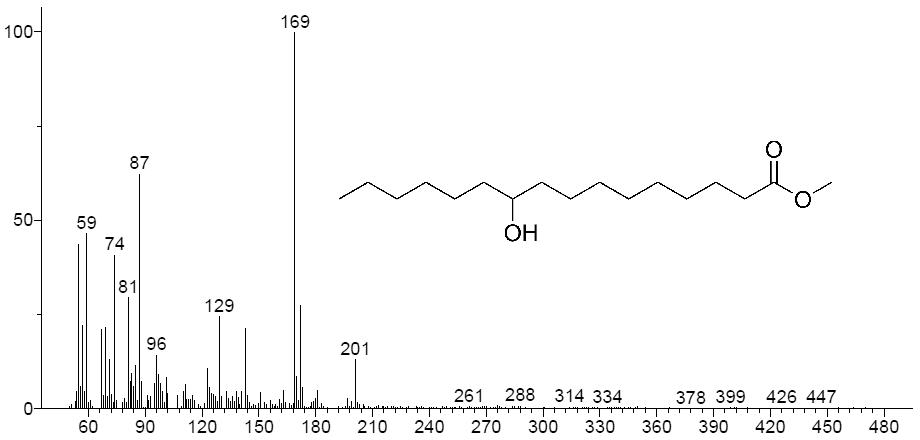


Mass spectrum of 10-hydroxyhexadecanoic acid methyl ester (10-OH-C16)


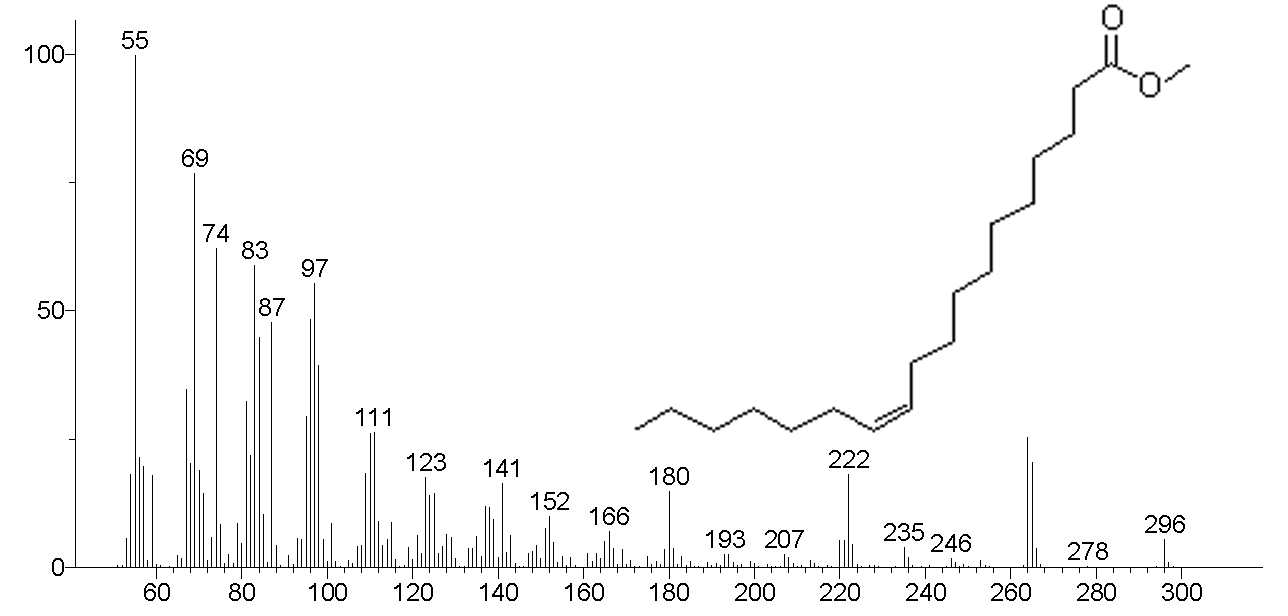


Mass spectrum of *cis*-vaccenic acid methyl ester (C18:1)


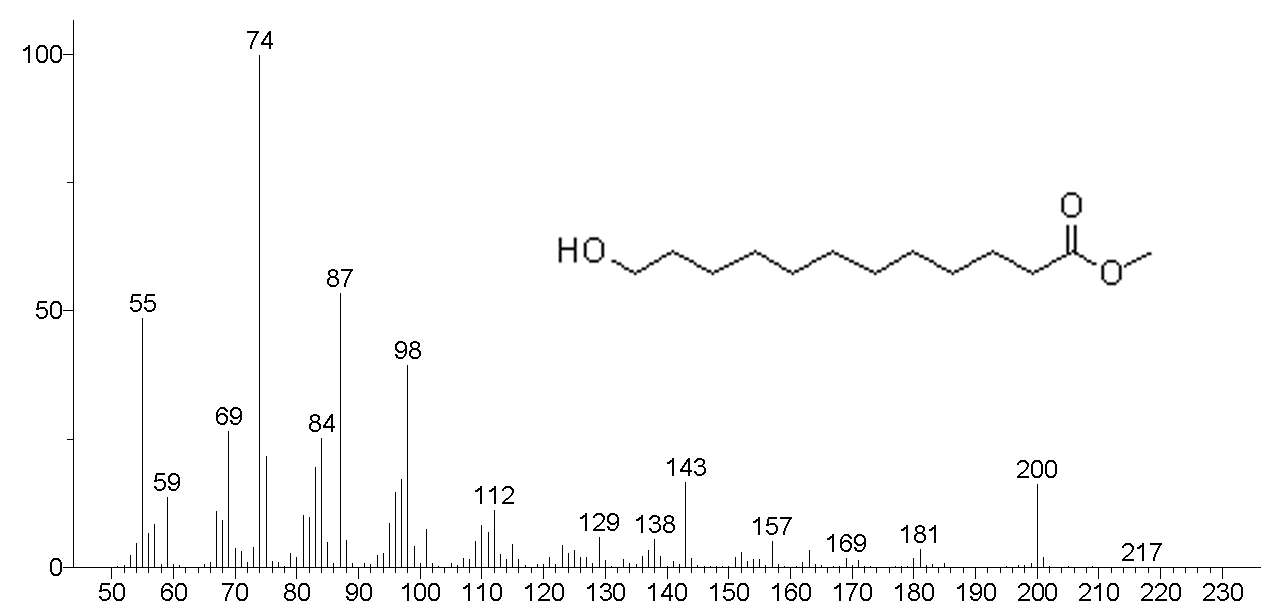


Mass spectrum of 12-hydroxydodecanoic acid methyl ester (12-OH-C12)


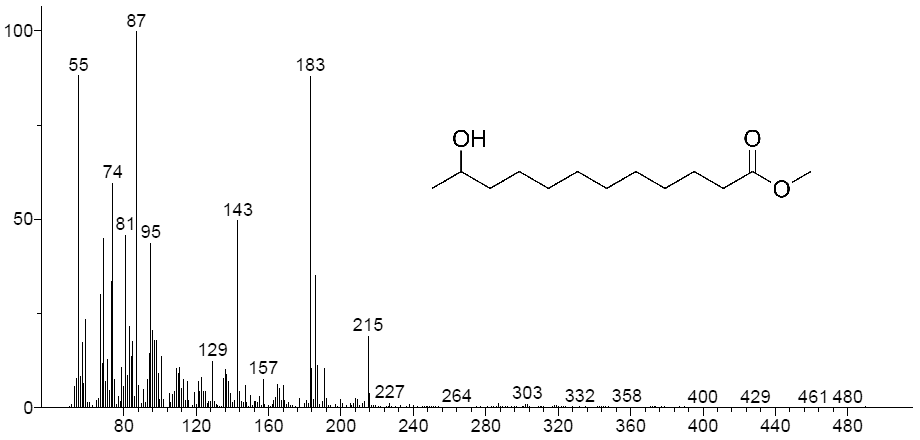


Mass spectrum of 11-hydroxydodecanoic acid methyl ester (11-OH-C12)


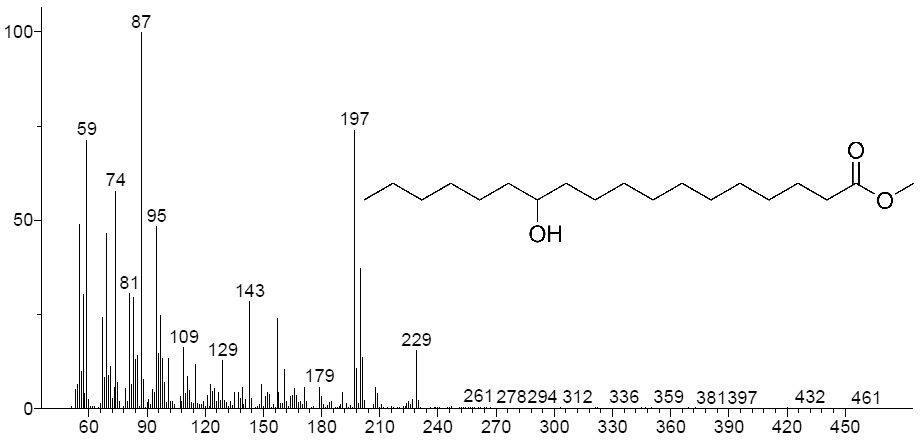


Mass spectrum of 12-hydroxyoctadecanoic acid methyl ester (12-OH-C18)


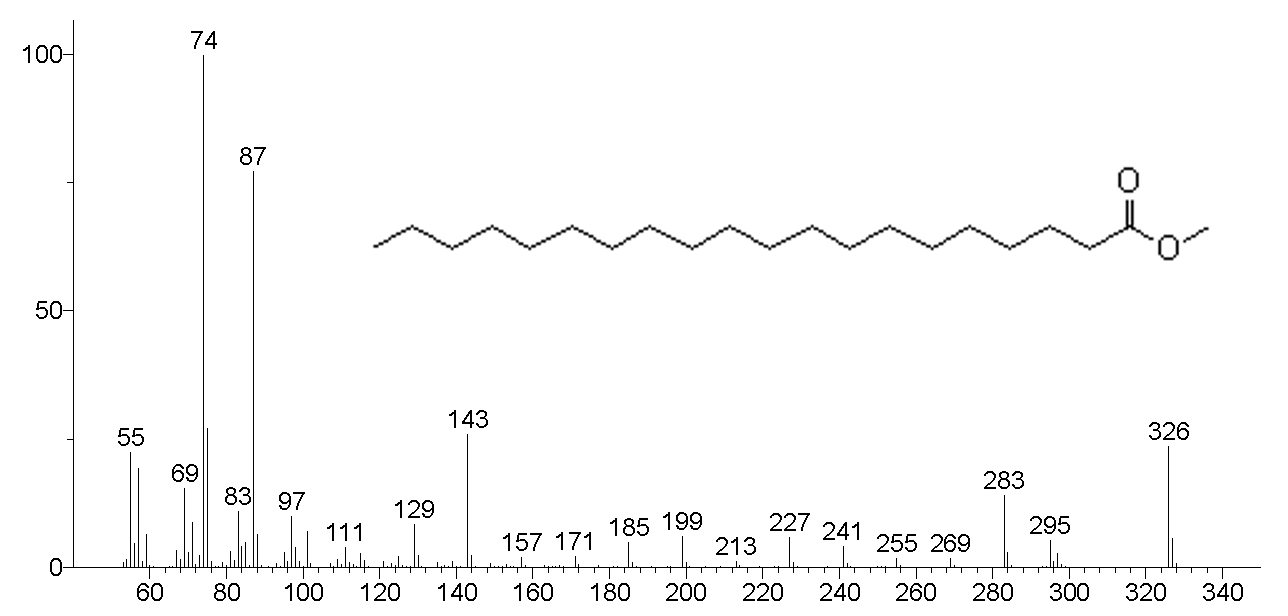


Mass spectrum of eicosanoic acid methyl ester (C20:0)
